# The Artificial Intelligence in Digital Radiology: *Part 2*: Towards an Investigation of *acceptance* and *consensus* on the Insiders

**DOI:** 10.3390/healthcare10010153

**Published:** 2022-01-14

**Authors:** Francesco Di Basilio, Gianluca Esposisto, Lisa Monoscalco, Daniele Giansanti

**Affiliations:** 1 Facoltà di Medicina e Psicologia, Sapienza University, Piazzale Aldo Moro, 00185 Rome, Italy; fdibasilio@hotmail.com (F.D.B.); gianluca.esposito.sapienza@gmail.com (G.E.); 2 Faculty of Engineering, Tor Vergata University, 00133 Rome, Italy; lisamonoscalco@hotmail.com; 3 Centre Tisp, Istituto Superiore di Sanità, 00161 Rome, Italy

**Keywords:** e-health, medical devices, m-health, digital-radiology, picture archive and communication system, artificial-intelligence, electronic surveys, chest CT, chest radiography, acceptance, consensus

## Abstract

*Background.* The study deals with the introduction of the artificial intelligence in digital radiology. There is a growing interest in this area of scientific research in *acceptance* and *consensus* studies involving both insiders and the public, based on surveys focused mainly on single professionals. *Purpose.* The goal of the study is to perform a contemporary investigation on the *acceptance* and the *consensus* of the three key professional figures approaching in this field of application: (1) Medical specialists in image diagnostics: the medical specialists (MS)s; (2) experts in physical imaging processes: the medical physicists (MP)s; (3) AI designers: specialists of applied sciences (SAS)s. *Methods.* Participants (MSs = 92: 48 males/44 females, averaged age 37.9; MPs = 91: 43 males/48 females, averaged age 36.1; SAS = 90: 47 males/43 females, averaged age 37.3) were properly recruited based on specific training. An electronic survey was designed and submitted to the participants with a wide range questions starting from the training and background up to the different applications of the AI and the environment of application. *Results.* The results show that generally, the three professionals show (a) a high degree of encouraging agreement on the introduction of AI both in imaging and in non-imaging applications using both standalone applications and/or *mHealth/eHealth*, and (b) a different consent on AI use depending on the training background. *Conclusions.* The study highlights the usefulness of focusing on both the three key professionals and the usefulness of the investigation schemes facing a wide range of issues. The study also suggests the importance of different methods of administration to improve the adhesion and the need to continue these investigations both with federated and specific initiatives.

## 1. Introduction

### Artificial Intelligence and Digital Radiology

The standardization of digital radiology caused important changes in the field of organ and functional diagnostics. This regards both the diagnostics and the interventional radiology [1,2]. It has led to exceptional changes in the organization of work and reporting processes. Furthermore, it pushed the digitization and computerization [3,4]. This solved and simplified many organizational problems, such as the organization of the archives, even if new ones appeared, such as those related to cybersecurity [5,6]. Today, digital radiology (DR) embraces a wide sector of diagnostic scenarios, also including sectors not directly related with the ionizing radiation, such as magnetic resonance and echography [7,8,9]. Those imaging sectors using DICOM are united under the hat of digital radiology [10,11,12,13]. Now, we are facing the possible impact of research on the health domain [14]. An important engine in this context is represented by the research efforts during the COVID-19 pandemic. For example, research on chest CT/radiography has opened important discussions and scenarios [15,16,17,18].

AI, a field of computer science [19], when used in the *health domain* is considered a tool able to perform tasks normally requiring human intelligence [20,21,22,23] that in recent years have been applied in various health-related areas, such as cancer detection [24], dementia classification [25], and drug design [26], to name a few.

If we consider the potential of AI in DR, the applications are multiple.

We need to consider four important *points of view* when we enter the field of DR [27,28]:

1. A *first point* of view is that DR includes different imaging sectors where it can potentially be applied. If we exclude imaging processes that do not involve ionizing radiation, we can identify the following sectors, both with reference to organ and total body diagnostics:
  - Interventional radiology
  - Diagnostic radiology (radiology, CT)
  - Nuclear magnetic resonance
  - Positron emission tomography
  - amma chamber
2. A *second point of view* is represented by the transversal sectors that embrace these disciplines in which AI can play an important role:
  - Therapy
  - Prevention
  - Quality control
  - Risk assessment
3. A *third point of view* is represented by the AI app distribution methods. In fact, we must not forget that AI, in the context of DR, has a future of standardization related to software for medical devices [29]. This software has different implications if it is used *standalone* or on the network, and if it is networked through *eHealth* or *mHealth* solutions. The implications also concern important aspects of cybersecurity [30].
4. A *fourth point of view* is represented by the specific training that must include AI and also the related disciplines such as informatics, medical imaging and the technologies for biomedical app.

**Figure 1:**
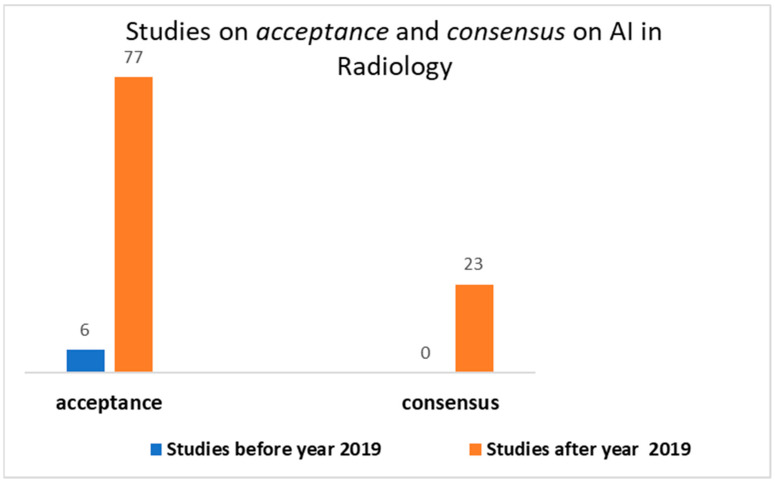
Output of the search on PubMed on acceptance and consensus on AI in radiology.

The passage of the AI into the routine of the DR (including the above listed *points of view*) must take place through an approach that provides for the transfer of evidence-based medicine (EBM) to the operational processes of the *health domain,* using all the available agreement tools, which include guidelines [31], technology assessment (TA) such as HTA and CER [32], and consensus conferences [33]. The latest definition of EBM, by Eddy [34], also considers the development of evidence-based policies in a multi-dimensional space of the *health domain,* involving quality, acceptance, consensus, and cost-effectiveness analysis. All the agreement tools will therefore also be based, as in other disciplines, on the *acceptance and consensus* of both the insiders and the public who will help to express important positions. A PubMed search in this area with the two keys [35,36]:

(acceptance) AND (artificial intelligence [Title/Abstract]) AND Radiology)

(consensus) AND (artificial intelligence [Title/Abstract]) AND Radiology)

shows (Figure 1) 83 results, of which 77 from 2019 to today for the acceptance and 23 results for the consensus, all comprised from 2019 to today.

This means that acceptance and consensus have become a priority on this issue over the past two years. Among the emerging tools in this area, we find the surveys useful as sensors for stakeholders and managers in general. These surveys [37,38,39,40,41,42,43,44,45,46,47] focused on some of the actors that revolve around this area: radiologists, radiographers, primary care providers (PCP), students, and patients, that is, both on service providers and users, but also on subjects in training. The studies on patients [37,38,39] have highlighted the curiosity and non-opposition to these techniques, together with the need to create culture, the need to educate on the issue and the fear for the aspects of cybersecurity in integration with *eHealth* and *mHealth*. The students [47] showed curiosity and optimism but complained about a lack of adequate training and the need to integrate specific modules into the training programs. Openings towards these solutions have emerged from studies on *radiologists* and *radiographers* [41,42,43,44,45,46] accompanied by the strong desire to have an important role in future *work-flow* modification processes and adequate training. In almost all studies, with rare exceptions such as [39], free and non-standardized questionnaires were used through validation processes, indicating that scholars, at this historical moment, are relying on their creativity to create increasingly innovative and adaptive questionnaires. Instruments, such as the TAM, widely used in radiology were not used [48].

What emerges from these studies are the following *needs of deepening* for further study in the surveys. Many figures have been thought of, such as the PCP [40], but others have been neglected. No studies have been identified on the specialists of applied sciences of artificial intelligence systems. In rare cases, surveys have been carried out which involved several professional figures, such as in [44,45], which involved both *radiologists* and *radiographers*. Our hypothesis is that the AI acceptance survey in radiology:

- must consider the above-listed (1–4) points of views, not limited to imaging and including the integration into *eHealth* and *mHealth* [49];
- must consider all the *involved professionals who have different training and a different work-flow and therefore different expectations from AI*.

Some studies on the design and test of AI solutions are clearly highlighting the importance of the team [50]. This team comprehends *(with a natural osmosis of skills)*:

- the medical physics;
- the medical specialist;
- the specialist in applied sciences.

**Figure 2:**
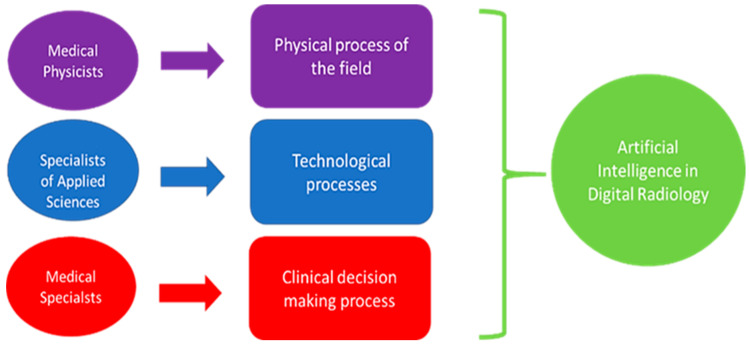
Interconnection among experts and AI.

A preparatory and preliminary step to the introduction of the AI in the clinical practice should directly face the *consensus/acceptance*. It emerges, based on the above, that important actors are undoubtedly (Figure 2): *medical specialists (MS)s*, *medical physicists (MP)s*, and *specialists of applied sciences (SAS)s*. MSs are a strategic role in the decision flow. MPs control the physical process. SASs design and maintain the AI tools (such as the biomedical engineers and technicians/technologists of radiology). The purpose of the study was: (a) to focus on these three professionals to investigate their *acceptance and consensus*; (b) to design and submit them a properly electronic survey for the investigation, with a wide range of features considering the highlighted needs of deepening the points listed above.

## 2. Methods

In line with the aim of the study, we decided to develop a survey.

The methodology comprehended: (I) the choice of the tool for the design of the electronic survey and (II) the adequacy to regulations; (III) the design of the survey based on the chosen tool respecting the wide range features to investigate; (IV) the dissemination on a population; (V) the data analysis based on an effective statistical approach. The questionnaire was developed using Microsoft Forms. It adhered to the SURGE Checklist [51] for the development and administration of the survey. The statistics followed two steps:

- Verification of data normality;
- Application of the ANOVA with a P lower than 0.01 for the significance of differences.

For the statistical confidence interval, we set a goal of 95%.

We considered that, among the most used tests to verify the normality, there are: (a) the Shapiro–Wilk test, which is preferable for a small sample; (b) the Kolmogorov–Smirnov test, which is instead used for more numerous samples. The samples in this study are small; therefore, we used the normality test of Shapiro–Wilk. We focused on the key figures (Figure 2) for the investigation.

**Figure 3:**
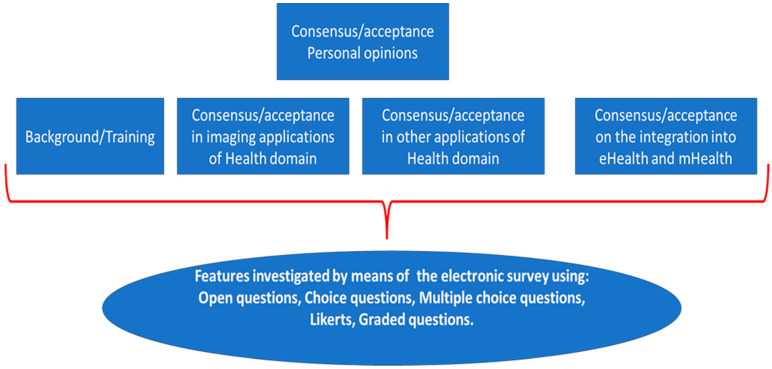
Features investigated by means of the electronic survey.

The electronic survey was designed to face a wide range of features (starting from the training and the background, up to the application of the AI and the environment of application) using: *choice questions, open questions, graded questions, and Likert* (Figure 3).

Both in the graded questions and in the Likert questions we fixed a six-level psychometric scale; it was therefore possible to assign a minimum score of one and a maximum score of six with a theoretical mean value (*TMV*) of 3.5. The *TMV* can be referred to for comparison in the analysis of the answers. An average value of the answers below TMV indicates a more negative than positive response. An average value above *TMV* indicates a more positive than negative response. The survey was accompanied by a brief description of the topic that would be addressed, clearly illustrating that the focus was related to the introduction of AI in digital radiology.

For the recruitment, we considered the three figures as planned, who, we remember, are medical specialists (MS), medical physicists (MP), specialists of applied sciences (SAS). All figures have a different role with AI in DR; this implies a different vision/opinion/consensus. The recruitment of these figures was very complex given that they belong to very different sectors, to different scientific societies. Currently, in Italy, there are 334 scientific societies [52]. We followed two paths that we have traced:

*First way*

In Italy, there are also federations of scientific societies that favor a scientific osmosis between the various scientific societies.

As regards the three professionals, we referred to:

- FEDERATION OF ITALIAN MEDICAL-SCIENTIFIC SOCIETIES [53] (includes associations such as the Italian association of medical and health physics and other relevant scientific societies and other societies operating in the Medical Diagnostics and in related fields) mainly for the first two professionals MPs and MSs but also for the SASs.
- FEDERATION OF SCIENTIFIC AND TECHNICAL ASSOCIATIONS [54] (contains the National Group of Bioengineering and other relevant scientific societies) and FEDERATION OF SCIENTIFIC ASSOCIATIONS OF RADIOLOGY TECHNICIANS [55] (contains for example the Italian association of system administrators and telemedicine, association of interventional radiology technicians, Health Imaging Sciences Association, and other relevant societies) mainly for the SASs but also for the other professionals.

It was possible for us to have lists of congresses in which to collect preliminary adhesions of interest for the project, in the presence, with contacts, encounters, discussions. A WhatsApp group was created to which the invitation and the anonymous questionnaire were sent, with a brief description and a recall of the discussion. In this way, it was possible to send the survey anonymously.

*Second way*

Sending was also carried out through our networks of WhatsApp, also following a peer-to-peer mechanism.

Table 1 reports the participants, the participants agreeing to continue after opening the questionnaire, and the related demographic characteristics. The average age of those who filled out the survey was not high. This depends on the very innovative and recent typology of the proposed theme, which was more attractive and inclusive (due to the training received) for the younger population.

**Figure A1:**
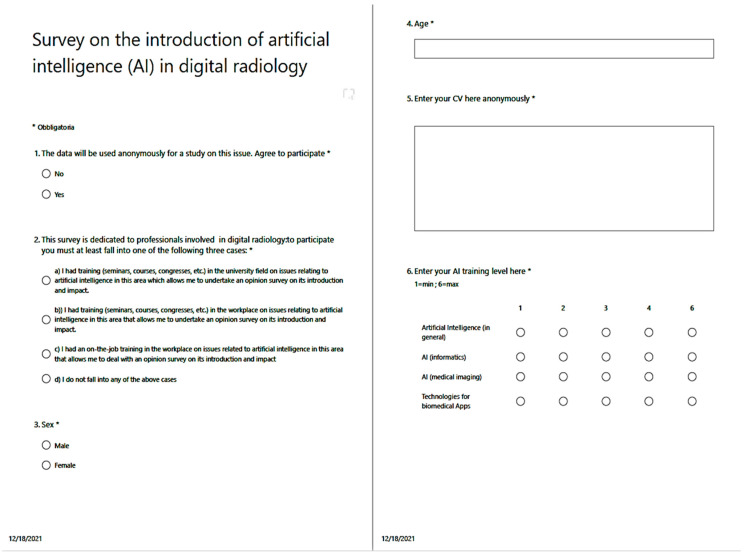
An example of the survey (first print screen).

**Figure A2:**
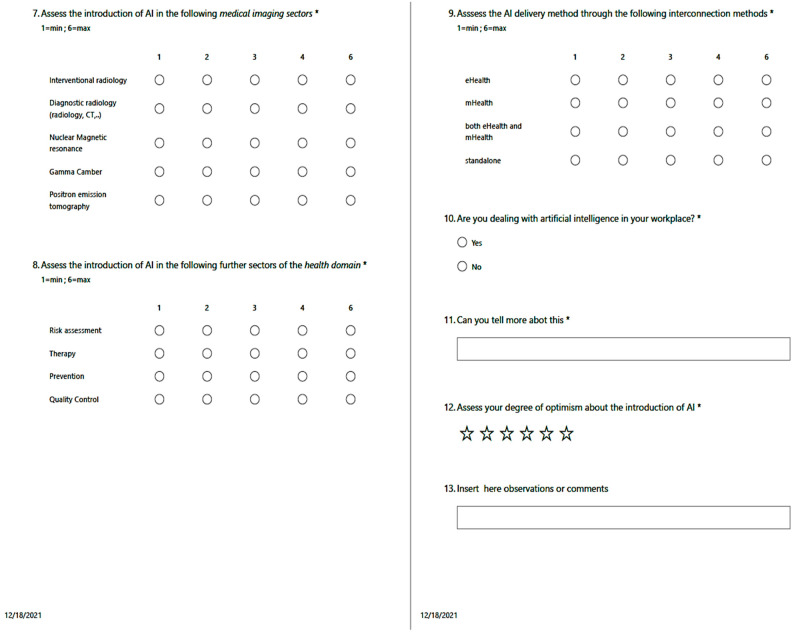
An example of the survey (second print screen).

Figure A1 and Figure A2 in the Appendix A show a sample of the questionnaire. It was converted from the Italian language into the English language.

## 3. Results

### 3.1. Outcome of the Closed Questions from the Survey

The eS contained a specific question relating to an adequate level of knowledge on AI to participate (through the attendance, for example, of specific academic and/or post-academic training). Only those who passed this requirement were admitted to the study. The results are organized into five tables. The first table (Table 2) concerns the *training on AI* aspects.

The second table (Table 3) concerns the consent/opinion on the application of AI specifically related to *medical imaging*.

The third table (Table 4) concerns the consent/opinion on the application on other medical aspects not directly related to medical imaging (*therapy, risk analysis, quality control, prevention*).

The fourth table (Table 5) concerns aspects on how it is considered convenient to approach AI regarding the information available (*eHealth, mHealth*, *Standalone,* both *eHealth* and *mHealth*) [43].

Table 6 reports the output on a graded question related on the generalized optimism related to the general use of AI.

Data were successfully preliminarily tested for the normality using the Shapiro test.

With regards to the *training* (Table 2), the subjects passing the barrier showed a high degree (score > TMV) in the three groups. However, the behavior was different in some cases. The ANOVA test highlighted some differences dependent on the different background: (a) in the case of *informatics*, where the SAS recorded a higher score; (b) in the case of *technologies for biomedical apps*, where both MPs and SASs showed a higher score.

We also included open-ended questions to investigate whether participants had direct experience (i.e., *training on the job*) in AI applied to the clinic. As far as MS is concerned, this can be represented, for example, by a direct experience of the clinical decision supported by AI. As for the MPs and SAS, this can be represented by direct activity on equipment equipped with AI systems as regards activities that can go from development to calibration and/or quality control. From these open questions, after classification and categorization, we found that a small percentage of respondents said they had or have such direct experience. A total of 14.3% of the MSs, 13.9% of the MPs, and 14.8% of the SASs had direct experience *of training on the job*. The *trained on the job* individuals showed a higher value of general optimism in the use of AI, uniform for the three groups (Table 6). With regards to the applications in medical imaging (Table 3), the subjects passing the barrier showed a high degree (score > TMV) in the three groups. The behavior was uniform. The ANOVA test highlighted no differences in all the issues among the groups. It is here evident that even if the background is different—*the MSs faced the diagnostic more; the MPS faced the imaging processes more; the SASs faced the technologies more*—the diversity compensated among themselves.

With regards to the use of AI in applications in the general fields (excluding the medical imaging) (Table 4), the subjects passing the barrier showed a high degree (score > TMV) in the three groups. However, the behavior was different in some cases. The ANOVA test highlighted some differences, dependent on the different background: (a) in the case of the more medical issues, *risk assessment*, *therapy*, and *prevention* where the MSs recorded a higher score; (b) in the case of *quality control*, both the MPs and SAS showed a higher score in this issue that is most related to the specific background. The opinion on the way of using/providing the AI (Table 5) is reported in consideration of the importance of the integration into the *eHealth* and *mHealth* [49]. With regards to this issue, the subjects passing the barrier showed a high degree (score > TMV) in the three groups, with a preference for the standalone approach. The preference for the standalone is probably due to the awareness on the exposition to the cyber risk. However, the behavior was different in some cases, where the SAS showed a lower score for the issues *mHealth, eHealth,* and *both.* This relates to the higher training in *informatics* (see above) that leads to higher awareness on the cyber risks when not applying AI in standalone.

### 3.2. Key Considerations from the Submission Process and Suggestions from the Open Questions

#### 3.2.1. Adhesion to the Survey

This type of administration will be more and more widespread in the future. Analyzing the peculiarities and the outcome of the recruitment mechanisms is therefore of primary importance. The two administrations took place in different time intervals to allow the evaluation of the contributions to the total data collection. Two paths were followed in our study. The first one began in 2019 with the collection of availability in presence at congresses with the possibility of an oral interaction/discussion and subsequent sending with WhatsApp.

**Figure 4:**
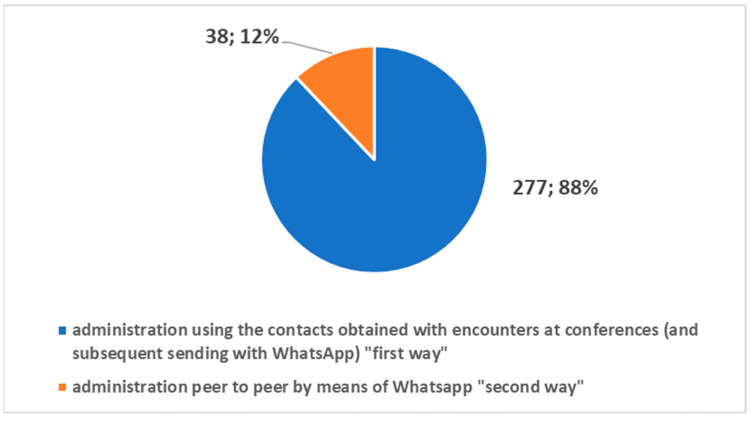
Contributions to the survey by the two different methods.

**Figure 5:**
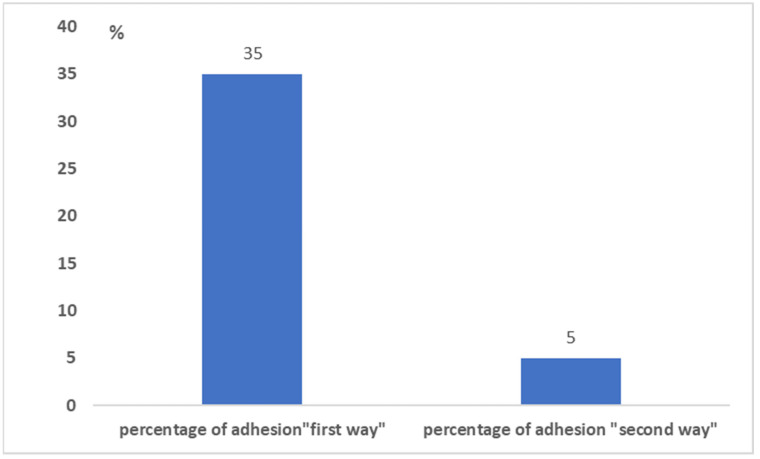
The percentage of adhesion to the survey by the two different methods.

The second was without oral discussion and was based on peer-to-peer sending via WhatsApp. Figure 4 highlights how the greatest contribution to data collection came through the first method based on (traditional) oral communication. Figure 5 shows the percentages of adhesions with respect to each method. The results show that the first method had a surprisingly higher percentage of adhesion. This demonstrates how the oral communication made of the three verbal, para-verbal, and non-verbal components continues to maintain a greater grip than a communication made with chat only.

#### 3.2.2. Outcome from the Open Question

In the survey to question No. 13, we optionally offered the possibility of reporting comments and observations.

Twenty-one interviewed people reported an observation or comment. We analyzed the comments that highlighted critical issues and suggestions for improvement, and we carried out datamining, which was followed by categorization.

**Figure 6:**
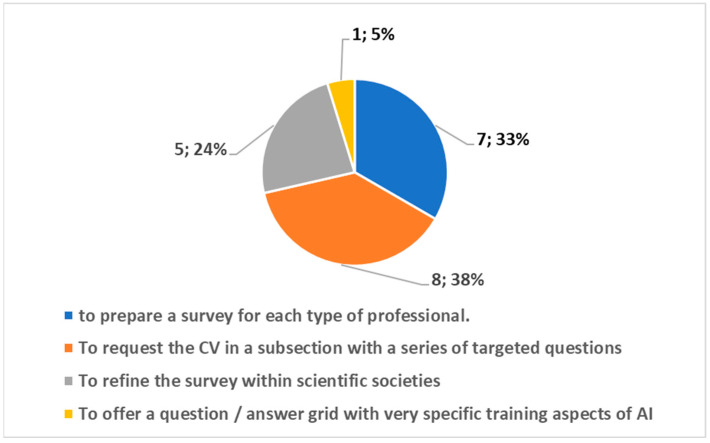
Suggestions for improvement with the obtained frequency of occurrence.

Figure 6 reports the following points as important suggestions for improvement based on the order of the frequency of occurrence:

o to request the CV in a subsection with a series of targeted questions;
o to prepare a survey for each type of professional;
o to refine the survey within scientific societies;
o to offer a question/answer grid with very specific training aspects of AI.

## 4. Discussion

We are undoubtedly about to face another important change in the world of digital radiology [14]: the introduction of AI in clinical practice. During the pandemic, the importance and potential of AI clearly emerged in two sectors of digital radiology: chest CT and chest radiography [15,16,17,18,43]. However, even before the pandemic, we were already talking about this phenomenon affecting the *health domain,* especially the sectors where the conversion to *digital health* has been heavy, such as the DR [27,28], thanks to the DICOM standardization process. DICOM is the container of the information arranged into pixels and/or voxels after the process of image acquisition. The pixels and/or voxels used as AI input carry different information of the investigated biomedical target. The information in those elements is related to the *physical process* (X-rays, gamma rays, magnetic fields, ultrasounds, etc.). Three elements play an important role: (1) the *physical process (PP)*, which depends on the physical fields used (X-rays, magnetic field, ultrasound, etc.); (2) the *technological process (TP)*, which concerns both the technologies for capturing information starting from the physical process, and the software implementation of AI-based algorithms; (3) the *decision-making process (DP)*, which must consider the outcome from the TP based on a PP and the human decision based on *medical knowledge* functionally related to both the TP and PP.

*It is for this reason that it is important that the experts of the DP, TP, and PP work are connected in the process of AI introduction and in the related investigations.*

It should also be borne in mind that in addition to diagnostic imaging, other AI applications used for *categorization into non-imaging* problems [9,27] (non-imaging categorization) were considered in the study. These range from risk analysis up to quality control. We also found it important to consider in the study how AI is delivered, whether it is delivered in standalone mode, or based on *mHealth* or *eHealth* [49]. In light of what has been illustrated above, we have decided to consider in the study the three figures of MSs, MPs, and SASs connected to (1,2) to investigate the *consensus and acceptance* by means of an eS. From a general point of view, these three professional figures showed a high degree of acceptance of the introduction of AI both in imaging and in non-imaging applications, using both standalone and network modes (*mHealth* or *eHealth*). Specifically, through a statistical assessment based on ANOVA, we were able to see a different way of approaching AI. This approach was uniform when considering AI applied to imaging. The approach was not uniform when considering the non-imaging applications and the delivery methods. Subjects with a background comprehending direct training on the job focused on AI showed the highest optimism. From a general point of view, the study highlights the usefulness of investigating the inclusion of AI through an eS, the usefulness of doing so based on three categories of experts (MSs; MPs; SASs), and the general optimism in the introduction of AI in digital radiology.

The background plays an important role in relation to the approach to AI. In the scientific literature, various studies already involved radiologists (key figures in the clinical decision) to perform reader studies. In a certain sense, if we look at the study proposed on a direct application of AI [50] in its entirety, we realize that regarding the enhancement of AI, the study we have proposed is in a complementary position. Our study directly focuses to the three involved professionals, having an active role in the flow from the tool design up to the decision [50]. Our study is in line with the studies based on surveys [37,38,39,40,41,42,43,44,45,46,47]; the submission of original surveys allows to obtain strategic information. In addition to similar studies, our study addressed the innovation of submitting the same survey to three key figures operating in the TP, PP, and DP. Furthermore, considering the needs that emerged from previous studies, our study proposed different survey schemes based on Likert/graded questions at six psychometric levels to have different quantitative outcomes, useful for categorizations.

A first scheme dealt with the educational, academic, and post-academic training aspects on modules relevant to the knowledge bases useful in this field.

A second scheme addressed the imaging aspects in detail, focusing on the different compatible DICOM tools used in DR.

The third scheme addressed the aspects of AI external to imaging but always relevant to the *work flow* (quality control, risk assessment, therapy and prevention) [27,28].

A fourth scheme was dedicated to integration with *eHealth and mHealth* [49], strategic for addressing important aspects such as cybersecurity.

From a general point of view, the study differs from other initiatives in this direction [56,57,58,59]. Furthermore, it offers to the scholars a complementary contribution and therefore complementary results if compared to study based on surveys [58,59]. Our proposed survey (see Appendix A) comprehends 13 questions (23 if we consider that the Likert has submodules): (a) it is oriented to all three professions potentially involved, (b) it goes into detail in the application of AI in the different sectors of imaging with a specific Likert and by means of another Likert in the application of AI in the translational sectors of the *health domain*, and (c) it addresses aspects of network integration (standalone, mHealth, eHealth) important for the impact on software medical device and cybersecurity. We have used several modules detailing the *choice questions, the open questions, the open large questions, and two modules used to give a psycho/sociometric assessment* (now currently used in the life sciences): the *graded questions and the Likert*. In addition, in our survey, there was also the possibility of supplementing the demographic information (including training) and work activity with two specific open questions, one *open large question* dedicated to the insertion of the CV, and one *open question* dedicated to the description of one’s own working activity.

The two surveys in [58,59] are in turn complementary; they are each dedicated to a specific professional figure and with different focuses.

The survey reported in [58] concerns a national audience, is focused on the MSs, and is made up of 13 questions: 4 dedicated to demographic aspects (age, region, activity, position and job site), 3 dedicated to interaction with AI (tasks by AI, advantages, issues), 3 dedicated to implications (ethical problems, risk of job loss, needs of policies), and other questions in complement, such as the opinion on the definition of AI.

The other survey [59] concerns an international audience, is dedicated to the figure of the MP, and consists of 25 questions. The first eight deal with the training aspects, the involvement in AI projects and with the activities and the opinion on the introduction of AI. Questions 9 to 17 all concern the collection of educational interests in a specific way and the opinions on the integration of the CV in future activities. An open question (number 18) is free. The final questions are all focused on demographics.

Our survey was submitted through two channels, both electronic (one of which, however, was also based on a preliminary in-person presentation of the initiative), which were evaluated. Part of the analysis was dedicated to the observations and criticalities that emerged, as well as specifically collected.

Both the surveys reported in [58,59] were administered with purely electronic methods, and there was no comparison between different modalities. They did not use graded questions and Likert questions. Furthermore, the critical issues to be addressed to improve these initiatives were not collected from both the surveys.

As regards the dissemination of the survey, our study shows that a preliminary contact in presence (followed by an electronic transmission) improves the participation rate. This suggests for the future to address these initiatives by preceding them by preliminary face-to-face meetings (for example, in focus groups or congresses). Regarding suggestions for improvement and development, it should be noted that those proposals that have had a frequency greater than 1 push towards a structured request in a grid of the CV, a specialization of the survey for the different professionals, and a refinement in scientific societies.

Considering these observations and what has emerged, the continuation of these initiatives in both a specialized and federated way is certainly desirable. It is hoped that the AI will be an opportunity to give birth to scientific federations that allow for in-depth initiatives in both a specific and confederate way.

## 5. Conclusions

The introduction of AI into clinical practice is now an unstoppable process that will take this discipline from research to routine use. Many professionals from now to the future will be involved, and it will be necessary to provide for targeted consensus actions to issue appropriate recommendations. Guidelines, TA reports, and consensus conferences, spread by scientific societies in the sector, for example, will in the future also use approaches based on surveys that scholars are currently developing.

Initiatives aimed at creating position papers in this area will be more and more frequent and will involve more and more teams of professionals, as in [56], where *medical physics* and *radiologists* have worked. Both national [57,58] and international [59] scientific societies could play an important role in the improvement and dissemination of these surveys, which could play a strategic role in monitoring the topic. It will also be important that scientific societies representing the different actors work as a team in initiatives that could possibly lead to stable and standardized international monitoring actions.

## Figures and Tables

**Table 1:** Characteristics of the participants in the study and the final involvement.

	Participants	Participants Agreeing to Continue/Passing the Requirement	Males/Females	Min Age/Max Age	Mean Age
*MSs*	111	108/92	48/44	32/43	37.9
*MPs*	105	97/91	43/48	31/41	36.1
*SASs*	99	93/90	47/43	33/40	37.3

**Table 2:** Specific outcome of the *perceived training.*

Knowledge	*MSs* *Score*	*MPs* *Score*	*SASs* *Score*	*ANOVA* *p*
**AI (general)**	4.56	4.38	4.51	***p* > 0.1**
**AI (informatics)**	4.33	4.24	**5.22**	***p* < 0.01**
**AI (medical imaging)**	4.98	5.07	5.02	***p* > 0.1**
**Technologies for biomedical Apps**	4.32	**5.03**	**5.11**	***p* < 0.01**

**Table 3:** Specific outcome of the opinion on the application *on the medical imaging.*

Application of AI in:	*MSs* *Score*	*MPs* *Score*	*SASs* *Score*	*ANOVA* *p*
	4.26	4.18	4.11	***p* > 0.1**
**Interventional radiology**	4.54	4.39	4.41	***p* > 0.1**
**Diagnostic radiology (radiology, CT, etc.)**	4.26	4.28	4.31	***p* > 0.1**
**Nuclear magnetic resonance**	4.61	4.69	4.72	***p* > 0.1**
**Positron emission tomography**	4.53	4.38	4.52	***p > 0.1***
**Gamma chamber**	4.44	4.39	4.43	***p > 0.1***

**Table 4:** Specific outcome of the opinion on the application of AI different from imaging.

Application of AI (Non Imaging)	*MSs* *Score*	*MPs* *Score*	*SASs* *Score*	*ANOVA* *p*
**Risk assessment**	**4.82**	4.21	**4.13**	***p* < 0.01**
**Therapy**	**5.21**	4.65	4.52	***p* < 0.01**
**Prevention**	**5.11**	4.02	4.11	***p* < 0.01**
**Quality Control**	4.12	**5.07**	**5.12**	***p* < 0.01**

**Table 5:** Specific outcome of the opinion on the use/delivery of the AI.

Scheme	*MSs* *Score*	*MPs* *Score*	*SASs* *Score*	*ANOVA* *p*
eHealth	4.72	4.66	**3.93**	***p* < 0.01**
mHealth	4.55	4.62	**3.89**	***p* < 0.01**
Both eHealth and mHealth	4.58	4.62	**3.86**	***p* < 0.01**
Standalone	5.33	5,24	5.17	***p* > 0.1**

**Table 6:** Optimism on the AI use.

Optimism	*MSs* *Score*	*MPs* *Score*	*SASs* *Score*	*ANOVA* *p*
**AI (All)**	4.58	4.57	4.53	***p* > 0.1**
**AI (people dealing with AI in the workplace)**	4.98	4.96	4.93	***p* > 0.1**

## Data Availability

Not applicable.

## Abbreviations

**Acronym**	**Description**
AI	Artificial intelligence
CT	Computerized tomography
MP	Medical physicist
SAS	Specialists of applied sciences
MS	Medical specialist
DICOM	Digital imaging and communications in medicine
DR	Digital radiology
TA	Technology assessment
HTA	Health technology assessment
CER	Comparative effectiveness research
PCP	Primary care provider
TP	Technological process
TMV	Theorical mean value
PP	Physical process
DP	Decision-making process
ANOVA	Analysis of variance
eHealth	Electronic health
mHealth	Mobile health

## References

[1] Thrall J.H. (2007). Teleradiology. Part I. History and clinical applications. Radiology.

[2] Thrall J.H. (2007). Teleradiology. Part II. Limitations, risks, and opportunities. Radiology.

[3] Reponen J. (2010). Teleradiology—Changing Radiological Service Processes from Local to Regional, International and Mobile Environment.

[4] Wootton R. (1996). Telemedicine: A cautious welcome. BMJ.

[5] Giansanti D. (2017). Teleradiology Today: The Quality Concept and the Italian Point of View. Telemed. E-Health.

[6] Orlacchio A., Romeo P., Inserra M.C., Grigioni M., Giansanti D. (2013). Guidelines for Quality Assurance and Technical Requirements in Teleradiology.

[7] Ruotsalainen P. (2010). Privacy and security in teleradiology. Eur. J. Radiol..

[8] Giansanti D. (2017). Diagnostic Imaging and E-Health: Standardization, Experiences and New Opportunities.

[9] Giansanti D. (2019). Diagnostics Imaging and M-Health: Investigations on the Prospects of Integration in Cytological and Organ Diagnostics.

[10] Canadian Association of Radiologists (2008). CAR Standards for Teleradiology.

[11] American College of Radiology (2002). ACR Standard for Teleradiology.

[12] Teleradiology Merrian-Webster Medical Dictionary Online. www.merriamwebster.com/medical/teleradiology.

[13] Dicom, Digital Imaging and Communication in Medicine. https://www.dicomstandard.org/.

[14] Giansanti D. (2020). The Artificial Intelligence in Digital Pathology and Digital Radiology: Where Are We?. Healthcare.

[15] Alsharif M.H., Alsharif Y.H., Yahya K., Alomari O.A., Albreem M.A., Jahid A. (2020). Deep learning applications to combat the dissemination of COVID-19 disease: A review. Eur. Rev. Med. Pharmacol. Sci..

[16] Ozsahin I., Sekeroglu B., Musa M.S., Mustapha M.T., Uzun Ozsahin D. (2020). Review on Diagnosis of COVID-19 from Chest CT Images Using Artificial Intelligence. Comput. Math. Methods Med..

[17] Pham T.D. (2020). Classification of COVID-19 chest X-rays with deep learning: New models or fine tuning?. Health Inf. Sci. Syst..

[18] Liang H., Guo Y., Chen X., Ang K.L., He Y., Jiang N., Du Q., Zeng Q., Lu L., Gao Z. (2022). Artificial intelligence for stepwise diagnosis and monitoring of COVID-19. Eur. Radiol..

[19] Stevenson A. (2010). Oxford Dictionary of English.

[20] Hsiang C.W., Lin C., Liu W.C., Lin C.S., Chang W.C., Hsu H.H., Huang G.S., Lou Y.S., Lee C.C., Wang C.H. (2022). Detection of left ventricular systolic dysfunction using an artificial intelligence-enabled chest X-ray. Can. J. Cardiol..

[21] Tajik A.J. (2016). Machine Learning for Echocardiographic imaging: Embarking on another incredible journey. J. Am. Coll. Cardiol..

[22] Krittanawong C., Zhang H., Wang Z., Aydar M., Kitai T. (2017). Artificial intelligence in precision cardiovascular medicine. J. Am. Coll. Cardiol..

[23] Zhang J., Gajjala S., Agrawal P., Tison G.H., Hallock L.A., Beussink-Nelson L., Deo R.C. (2018). Fully automated echocardiogram interpretation in clinical practice. Circulation.

[24] Rodriguez-Ruiz A., Lång K., Gubern-Merida A., Broeders M., Gennaro G., Clauser P., Helbich T.H., Chevalier M., Tan T., Mertelmeier T. (2019). Stand-Alone Artificial Intelligence for Breast Cancer Detection in Mammography: Comparison With 101 Radiologists. J. Natl. Cancer Inst..

[25] Bertini F., Allevi D., Lutero G., Montesi D., Calzà L. (2022). Automatic Speech Classifier for Mild Cognitive Impairment and Early Dementia. ACM Trans. Comput. Healthc..

[26] Mak K.K., Pichika M.R. (2019). Artificial intelligence in drug development: Present status and future prospects. Drug Discov. Today.

[27] Jalal S., Nicolaou S., Parker W. (2019). Artificial Intelligence, Radiology, and the Way Forward. Can. Assoc. Radiol. J..

[28] European Society of Radiology (ESR) (2019). What the radiologist should know about artificial intelligence—An ESR white paper. Insights Imaging.

[29] Regulation (EU) 2017/745 of the European Parliament and of the Council of 5 April 2017 on Medical Devices, Amending Directive 2001/83/EC, Regulation (EC) No 178/2002 and Regulation (EC) No 1223/2009 and Repealing Council Directives 90/385/EEC and 93/42/EEC.2017. https://eur-lex.europa.eu/legal-content/EN/TXT/HTML/?uri=CELEX:32017R0745&from=IT.

[30] Giansanti D. (2021). Cybersecurity and the Digital-Health: The Challenge of This Millennium. Healthcare.

[31] Evidence-Based Medicine Guidelines. https://www.ebm-guidelines.com/dtk/ebmg/home.

[32] Luce B.R., Drummond M., Jönsson B., Neumann P.J., Schwartz J.S., Siebert U., Sullivan S.D. (2010). EBM, HTA, and CER: Clearing the confusion. Milbank Q..

[33] McGlynn E.A., Kosecoff J., Brook R.H. (1990). Format and conduct of consensus development conferences. Multi-nation comparison. Int. J. Technol. Assess. Health Care.

[34] Eddy D.M. (2005). Evidence-Based Medicine: A Unified Approach. Health Affairs.

[35] National Library of Medicine. https://pubmed.ncbi.nlm.nih.gov/?term=%28acceptance%29+AND+%28artificial+intelligence%5BTitle%2FAbstract%5D%29+AND+Radiology&sort=date&size=200.

[36] National Library of Medicine. https://pubmed.ncbi.nlm.nih.gov/?term=%28%28consensus%29+AND+%28artificial+intelligence%5BTitle%2FAbstract%5D%29%29+AND+%28radiology%5BTitle%2FAbstract%5D%29&sort=date&size=200.

[37] Lennartz S., Dratsch T., Zopfs D., Persigehl T., Maintz D., Hokamp N.G., Dos Santos D.P. (2021). Use and Control of Artificial Intelligence in Patients Across the Medical Workflow: Single-Center Questionnaire Study of Patient Perspectives. J. Med. Internet Res..

[38] Zhang Z., Citardi D., Wang D., Genc Y., Shan J., Fan X. (2021). Patients’ perceptions of using artificial intelligence (AI)-based technology to comprehend radiology imaging data. Health Inform. J..

[39] Ongena Y.P., Haan M., Yakar D., Kwee T.C. (2020). Patients’ views on the implementation of artificial intelligence in radiology: Development and validation of a standardized questionnaire. Eur. Radiol..

[40] Hendrix N., Hauber B., Lee C.I., Bansal A., Veenstra D.L. (2021). Artificial intelligence in breast cancer screening: Primary care provider preferences. J. Am. Med. Inform. Assoc..

[41] Abuzaid M.M., Elshami W., McConnell J., Tekin H.O. (2021). An extensive survey on radiographers from the Middle East and India on artificial intelligence integration in radiology practice. Health Technol..

[42] Abuzaid M.M., Tekin H.O., Reza M., Elhag I.R., Elshami W. (2021). Assessment of MRI technologists in acceptance and willingness to integrate artificial intelligence into practice. Radiography.

[43] Giansanti D., Rossi I., Monoscalco L. (2021). Lessons from the COVID-19 Pandemic on the Use of Artificial Intelligence in Digital Radiology: The Submission of a Survey to Investigate the Opinion of Insiders. Healthcare.

[44] Abuzaid M.M., Elshami W., Tekin H., Issa B. (2020). Assessment of the Willingness of Radiologists and Radiographers to Accept the Integration of Artificial Intelligence into Radiology Practice. Acad. Radiol..

[45] Alelyani M., Alamri S., Alqahtani M.S., Musa A., Almater H., Alqahtani N., Alshahrani F., Alelyani S. (2021). Radiology Community Attitude in Saudi Arabia about the Applications of Artificial Intelligence in Radiology. Healthcare.

[46] European Society of Radiology (ESR) (2019). Impact of artificial intelligence on radiology: A EuroAIM survey among members of the European Society of Radiology. Insights Imaging.

[47] Galán G.C., Portero F.S. (2021). Medical students’ perceptions of the impact of artificial intelligence in Radiology. Radiologia.

[48] Aldosari B. (2012). User acceptance of a picture archiving and communication system (PACS) in a Saudi Arabian hospital radiology department. BMC Med. Inform. Decis. Mak..

[49] Moss R.J., Süle A., Kohl S. (2019). eHealth and mHealth. Eur. J. Hosp. Pharm..

[50] Shan H., Padole A., Homayounieh F., Kruger U., Khera R.D., Nitiwarangkul C., Kalra M.K., Wang G. (2019). Competitive performance of a modularized deep neural network compared to commercial algorithms for low-dose CT image reconstruction. Nat. Mach. Intell..

[51] Moher D., Altman D.G., Schulz K.F., Simera I., Wager E. Guidelines for Reporting Health Research: A User’s Manual. https://onlinelibrary.wiley.com/doi/abs/10.1002/9781118715598.ch20.

[52] Ministero Della Salute Rivede Elenco Società Scientifiche per Stesura Linee Guida 41 Società in Più. http://www.aiponet.it/news/104-ufficio-stampa/2149-ministero-della-salute-rivede-elenco-societa-scientifiche-per-stesura-linee-guida-41-societa-in-piu.html.

[53] Federazione Delle Società Medico-Scientifiche Italiane. https://portale.fism.it/.

[54] Federazione Delle Associazioni Scientifiche dei Tecnici di Radiologia. https://www.associazionefaster.org/.

[55] Federazione Delle Associazioni Scientifiche e Tecniche. https://fast.mi.it/chi-siamo/.

[56] Thomassin-Naggara I., Balleyguier C., Ceugnart L., Heid P., Lenczner G., Maire A., Séradour B., Verzaux L., Taourel P. (2019). Conseil National Professionnel de la Radiologie et Imagerie Médicale (G4). Artificial intelligence and breast screening: French Radiology Community position paper. Diagn. Interv. Imaging.

[57] Avanzo M., Trianni A., Botta F., Talamonti C., Stasi M., Iori M. (2021). Artificial Intelligence and the Medical Physicist: Welcome to the Machine. Appl. Sci..

[58] Coppola F., Faggioni L., Regge D., Giovagnoni A., Golfieri R., Bibbolino C., Miele V., Neri E., Grassi R. (2021). Artificial intelligence: Radiologists’ expectations and opinions gleaned from a nationwide online survey. Radiol. Med..

[59] Diaz O., Guidi G., Ivashchenko O., Colgan N., Zanca F. (2021). Artificial intelligence in the medical physics community: An international survey. Phys. Med..

